# Aflatoxin B1 DNA-Adducts in Hepatocellular Carcinoma from a Low Exposure Area

**DOI:** 10.3390/nu14081652

**Published:** 2022-04-15

**Authors:** Laura Gramantieri, Federica Gnudi, Francesco Vasuri, Daniele Mandrioli, Francesca Fornari, Francesco Tovoli, Fabrizia Suzzi, Andrea Vornoli, Antonia D’Errico, Fabio Piscaglia, Catia Giovannini

**Affiliations:** 1 Division of Internal Medicine, Hepatobiliary and Immunoallergic Diseases, IRCCS Azienda Ospedaliero-Universitaria di Bologna, 40138 Bologna, Italy; francesco.tovoli@studio.unibo.it (F.T.); fabio.piscaglia@unibo.it (F.P.); 2 Cesare Maltoni Cancer Research Center, Ramazzini Institute, Via Saliceto 3, 40010 Bentivoglio, Italy; gnudif@ramazzini.it (F.G.); mandriolid@ramazzini.it (D.M.); andreaw86@hotmail.it (A.V.); 3 Pathology Unit, IRCCS Azienda Ospedaliero-Universitaria di Bologna, 40138 Bologna, Italy; francesco.vasuri@aosp.bo.it (F.V.); antonietta.derrico@unibo.it (A.D.); 4 Department for Life Quality Studies, University of Bologna, 40126 Rimini, Italy; francesca.fornari2@unibo.it; 5 Center for Applied Biomedical Research (CRBA), University of Bologna, 40138 Bologna, Italy; fabrizia.suzzi3@unibo.it; 6 Department of Experimental, Diagnostic and Specialty Medicine, University di Bologna, 40138 Bologna, Italy; 7 Department of Medical and Surgical Science, University of Bologna, 40138 Bologna, Italy

**Keywords:** AFB1, HCC, CD68, cholangiocarcinoma, food control

## Abstract

Aflatoxin B1 (AFB1) is a class 1 carcinogen with an ascertained role in the development of hepatocellular carcinoma (HCC) in high exposure areas. Instead, this study aimed to assay whether chronic/intermittent, low-dose AFB1 consumption might occur in low-exposure geographical areas, ultimately accumulating in the liver and possibly contributing to liver cancer. AFB1-DNA adducts were assayed by immunostaining in liver tissues from three Italian series of twenty cirrhosis without HCC, 131 HCC, and 45 cholangiocarcinoma, and in an AFB1-induced HCC rat model. CD68, TP53 immunostaining, and TP53 RFLP analysis of R249S transversion were used to characterize cell populations displaying AFB1-DNA adducts. Twenty-five HCCs displayed AFB1-adducts both in neoplastic hepatocytes and in cells infiltrating the tumor and non-tumor tissues. Nuclear immunostaining was observed in a few cases, while most cases showed cytoplasmic immunostaining, especially in CD68-positive tumor-infiltrating cells, suggestive for phagocytosis of dead hepatocytes. Similar patterns were observed in AFB1-induced rat HCC, though with higher intensity. Cholangiocarcinoma and cirrhosis without HCC did not displayAFB1-adducts, except for one case. Despite not providing a causal relationship with HCC, these findings still suggest paying attention to detection and control measures for aflatoxins to ensure food safety in low exposure areas.

## 1. Introduction

Aflatoxins (AF) are mycotoxins produced by *Aspergillus* fungi that contaminate food, particularly cereals, wheat, oilseeds, spices, nuts, milk, meat, eggs, maize, and peanuts, especially in certain geographical areas where food preservation is sub-optimal [1,2,3]. Occupational settings are also considered at risk for AFs exposure [4]. Ingestion of contaminated food implies the risk of either high or low exposure, and AF-induced liver damage may occur with an acute or chronic pattern. Carcinogenic effects are mainly associated with chronic exposure. The International Agency for Research on Cancer (IARC) introduced Aflatoxin B1 (AFB1) among class 1 carcinogens in 2012. AFB1 is one of the most potent carcinogens found in our food supply, and its role in the development of hepatocellular carcinoma (HCC) was demonstrated both in humans and animals [5,6]. Mechanistically, AFB1 is activated by CYP450 enzymes to its 8,9-epoxide isoform, which displays its carcinogenic effects by reacting with the N7 of guanine in RNA and DNA to form AFB1-N7-guanine (AFB1-N7-Gua) and two diastereomers of the imidazole ring-opened 8,9-dihydro-8-(2,6-diamino-4-oxo-3,4-dihydropyrimid-5-yl-formamido)-9-hydroxy AFB1 (AFB1-FapyGua). These events initiate carcinogenesis by epoxidation and DNA adduction, and work synergistically with hepatitis B virus [7] and hepatitis C virus or alcohol [8]. Hepatocytes are the liver cell lineage most exposed to AFB1 genotoxic damage, due to their constitutive expression of microsomal cytochrome P450 enzymes. At the molecular level, AFB1-adducts mostly lead to G → T transversions. Viral-chemical interactions and mechanisms underlying the molecular pathogenesis of AFB1-exposed cirrhosis and HCC were extensively investigated. The coexistence of HBV infection and AFB1 exposure was causally associated with TP53 R249S transversion in high exposure areas, providing molecular evidence corroborating epidemiological data [9]. Conversely, very few data are available concerning low and middle exposure countries. Despite being less relevant, AF exposure may not be negligible in geographical areas such as Italy, where climate changes favouring the growth of contaminating fungi and the globalization of flour and nut distribution may facilitate AFB1 spread. Remarkably, the extensive use of spices, tea, and herbal teas of Asian origin, together with unverified herbal medicine products and the globalization of food commerce, also put at possible risk those geographical areas assumed to be spared from AF contamination. Maize contamination by AF, for instance, was detected in Italy in 2003. It was likely the consequence of scarce rainfall and elevated temperatures that occurred during the 2003 maize season, leading to abnormal AF levels in cow milk [10]. According to EFSA [11], current levels of exposure to AFB1 derived from food raise health concerns. According to IARC [12], the capacity of a substance to produce DNA adducts is a key characteristic of carcinogens. It is thus conceivable that chronic/intermittent, low-dose AFB1 consumption with food and beverages might also occur in geographical areas assumed to be at low risk, ultimately accumulating in the liver. 

To test this hypothesis, we examined three Italian series of patients with cirrhosis without HCC, cirrhosis complicated by HCC, or cholangiocellular carcinoma (CC), to start elucidating the relevance of AFB1-DNA adducts in primary liver tumors in a geographical area that is assumed to be at low exposure and consequently, where AFB1 is not considered among the risk factors for HCC. In these patients, we investigated the presence of AFB1-DNA adducts in HCC, CC, or end-stage cirrhotic livers without HCC, to ascertain any association between AFB1-DNA adducts and HCC or CC. We also analysed an AFB1-induced HCC rat model to evaluate any morphological correlation with the human counterpart. Our results suggest considering the possible role of AFB1 exposure among risk factors contributing to HCC in Italy, and to pay attention to possible sources of AFB1 even in low-exposure geographical areas. 

## 2. Patients and Methods

### 2.1. Patients

Liver tissue from patients with end-stage cirrhosis submitted to transplantation, and tumor and surrounding tissues of resected HCC or CC were screened in our patient series and selected based on the availability of a formalin-fixed, paraffin-embedded (FFPE) tissue block and well annotated clinical-pathological information. All patients enrolled in the study lived in Italy and denied any prolonged staying abroad except for a few days of vacation that year in a few cases. Local ethics committee of St. Orsola-Malpighi University Hospital approved this study (138/2015/O/Tess). 

Three independent patient cohorts were examined:

1. Cohort 1: 131 surgically resected HCCs were studied by immunohistochemistry (IHC) to determine the presence of AFB1-adducts, mutated TP53 aberrant accumulation, and CD68, to characterize AFB1-adducts positive cells among the tumor-infiltrating cell populations. Restriction fragment length polymorphism (RFLP) analysis of TP53 R249S transversion was performed in a subgroup of 77 patients, while 27 matched HCC and cirrhotic tissues were previously sequenced to detect TP53 gene mutations [13].
2. Cohort 2: 45 surgically-resected CC were studied by IHC to determine the presence of AFB1-adducts.
3. Cohort 3: 20 explanted end-stage cirrhotic livers were studied by IHC to determine the presence of AFB1 adducts, mutated TP53 aberrant accumulation, CD68 IHC to characterize AFB1 adducts positive cells.

Characteristics of patients’ cohorts (gender, etiology of the underlying liver disease, smoking history) are described in Table 1.

### 2.2. Rat Model of HCC Exposed to AFB1

Liver tissue blocks belonging to a historical series of six Sprague-Dawley (SD) rats bearing both adenomas and HCCs following AFB1 exposure from the Ramazzini Institute (RI) were studied to ascertain any morphological correlation with human HCC. The experiments were conducted according to the Italian law regulating, at the time, the protection of animals used for experimental and other scientific purposes (Decreto Legislativo 116/1992). AFB1 was administered by gavage during the 6th, 7th, and 8th week after birth. Nine doses of 70 micrograms/rat dissolved in Dimethyl Sulfoxide (DMSO) were administered to each rat. Animals were sacrificed at 80 weeks (two cases), or they spontaneously died at 72–118 weeks (four cases). Three SD rats with HCC and one with cholangiocellular carcinoma developed after benzene exposure were also studied. Rats were exposed to benzene by inhalation at the concentration of 200 ppm, 4–7 h daily, for 14 weeks and then sacrificed at 53 weeks of age (three cases), or exposed for 27 weeks and then kept under observation until spontaneous death (one case). Finally, two SD rats spontaneously developing HCC, two spontaneously developing adenomas, and one spontaneously developing cholangiocellular carcinoma were also included into the study as a control group.

### 2.3. Immunohistochemical Assay of AFB1 Adducts

Aflatoxin B1 adducts were immunohistochemically assessed on formalin-fixed, paraffin embedded sections. Briefly, 4-µm-thick paraffin sections were rehydrated and inhibition of endogenous peroxidases activity was obtained by incubating slides in 3% H_2_O_2_–methanol for 20 min at 4 °C. Sections were then washed with 0.5 M glycine in phosphate-buffered saline (PBS) for 10 min and incubated at room temperature with 2% of normal goat serum for 30 min. Avidin/biotin blocking kit (Vector Laboratories, Burlingame, CA, USA) was used to block both biotin and avidin endogenous binding sites. Sections were incubated with a 1:100 dilution of anti-AFB1 antibody (monoclonal anti-AFB1 6A10-Biotin; Novus Biologicals Inc, Abingdon, UK) overnight at 4 °C. Secondary marking was performed with the Vectastain ABC kits (Vector Laboratories, Burlingame, CA, USA). Negative controls were obtained by omitting the primary antibody. The immunohistochemical staining was visualized using the diaminobenzidine colour substrate (Sigma, St Louis, MO, USA) followed by counterstaining with Mayer’s haematoxylin. Sections were dehydrated in alcohol, cleared in toluene, and mounted with DPX (Sigma, St Louis, MO, USA). Immunostaining was independently scored as negative (absence of positive cells), low (less than 10 cells), intermediate (10–40 cells), or high (more than 40 cells), based on the number of positive cells in 10 consecutive 40× magnification fields by two observers (LG and CG). Positive cell types (hepatocytes or tumor-infiltrating cells) were annotated. 

**TP53 immunostaining** was investigated to assay whether AFB1 adducts co-localised with mutated TP53. Abnormal TP53 protein accumulation was immunohistochemically assessed by using the clone DO-7, mouse anti-human p53 (DAKO, Glostrup, Denmark). Next, 4 μm thick sections were processed for haematoxylin and eosin staining and for immunohistochemistry. Endogenous peroxidases were inhibited by incubating slides in 3% H_2_O_2_–methanol for 20 min at 4 °C. Negative controls were obtained by omitting the primary antibody. Immunoreactivity was revealed with the EnVision system (DAKO, Glostrup, Denmark), and diaminobenzidine (DAB) as chromogen (Sigma, St Louis, MO, USA), and it was considered positive (corresponding to mutated TP53) when >10% neoplastic hepatocytes were strongly immunostained [14]. 

**Restriction fragment length polymorphism (RFLP)** analysis of TP53 R249S transversion was performed in a subgroup of 77 patients including all the 25 AFB1 positive HCCs and 52 negative cases. DNA was amplified by PCR for exon 7 of the p53 gene using specific primers: forward primer 5′-CTTGCCACAGGTCTCCCCAA-3′ and reverse primer 5′-AGGGGTCACCGGCAAAGCAGA-3′. Next, 10 μL of PCR products were digested with HaeIII restriction endonucleases (Promega, Milan, Italy) which cleaves GG/CC sequence between codon 249 and 250 to generate 66 bp and 92 bp fragments, while the presence of codon 249 mutation yields an undigested 158 bp fragment. Among the 77 cases tested by RFLP, 27 HCC tissues were previously sequenced to detect *TP53* gene mutations [13]. *TP53* exons 4–10, along with flanking intronic boundaries of the *TP53* gene [GenBank Reference No.NC_000017.10 (7571720–7590863); RefSeqGeneID NG_017013.1], were screened by WAVE denatured high-performance liquid chromatography (dHPLC, Transgenomic, San Jose, CA, USA) followed by direct sequencing, to characterize nucleotide variants on PCR products showing the presence of heteroduplexes (ABI PRISM 3730 Genetic Analyzer, Applied Biosystems, Monza, Italy). 

**CD68 immunostaining** was used to ascertain whether intra- and extra-tumoral AFB1 adducts positive cells might be ascribed to tumor-infiltrating monocyte-derived cells. Indeed, CD68 recognizes monocyte-macrophage cells, which in the liver are represented by the resident Kupffer cells and/or blood-derived macrophages. Immunohistochemistry for CD68 (clone PG-M1) was carried out by means of the immunostainer Benchmark^®^ ultra (Ventana Medical Systems, Inc., Roche group, Tucson, AZ, USA), following the manufacturer’s procedures.

### 2.4. Statistical Analysis

Associations between AFB1-DNA adducts in HCC tissue and demographic, etiologic, and molecular characteristics were analyzed by Chi-square test with Yates correction. Reported p-values were two-sided and considered significant when lower than 0.05. Statistical calculations were executed using SPSS version 20.0 (SPSS Inc., Chicago, IL, USA). * *p* < 0.05, ** *p* < 0.01. 

## 3. Results

### 3.1. AFB1 Adducts Are Present in HCC but Not in CC Tissues

**Figure 1:**
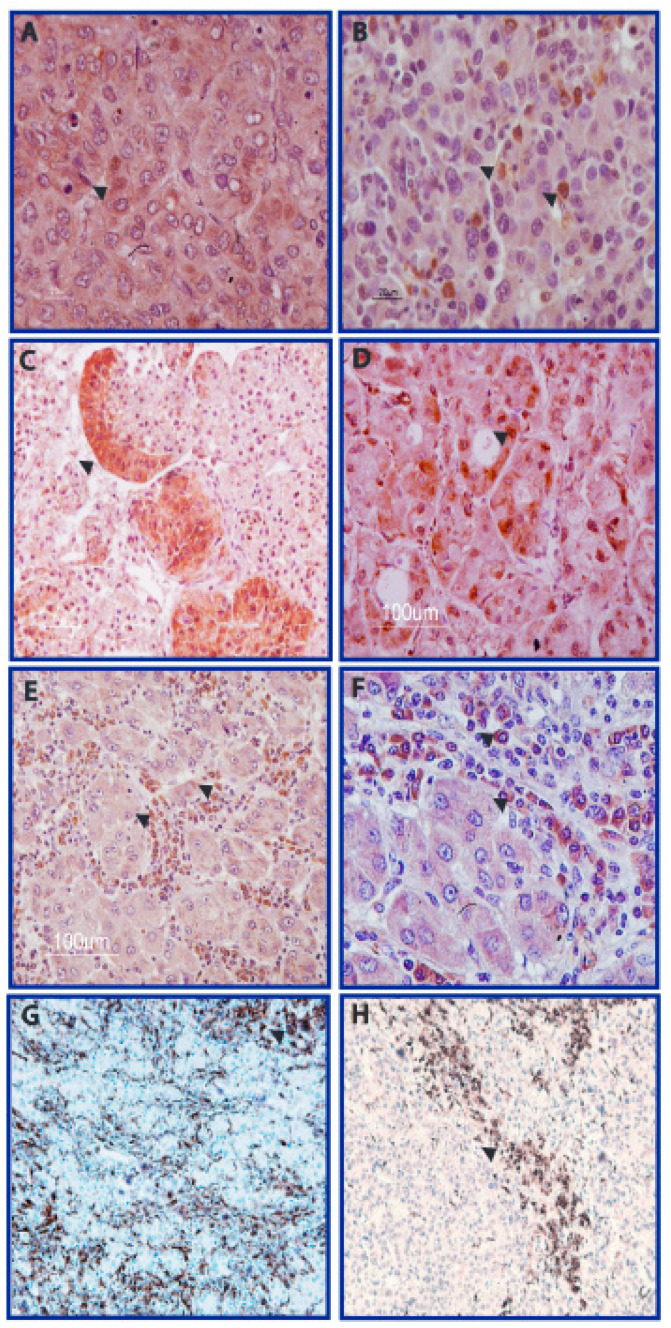
AFB1 staining in human neoplastic hepatocytes and in tumor-infiltrating cells. (**A**–**D**). Representative cases of AFB1-adducts immunostaining in neoplastic hepatocytes and tumor-infiltrating cells displaying a variable degree and extension. (**E**,**F**): Tumor-infiltrating cells positive to AFB1-adducts were further characterised by CD68 immunostaining (**G**,**H**), confirming their monocytic origin. Scale bars are reported. Magnification: (**A**,**B**): 40×; (**C**): 20×; (**F**): 40×; (**D**–**H**): 10×.

**Figure 2:**
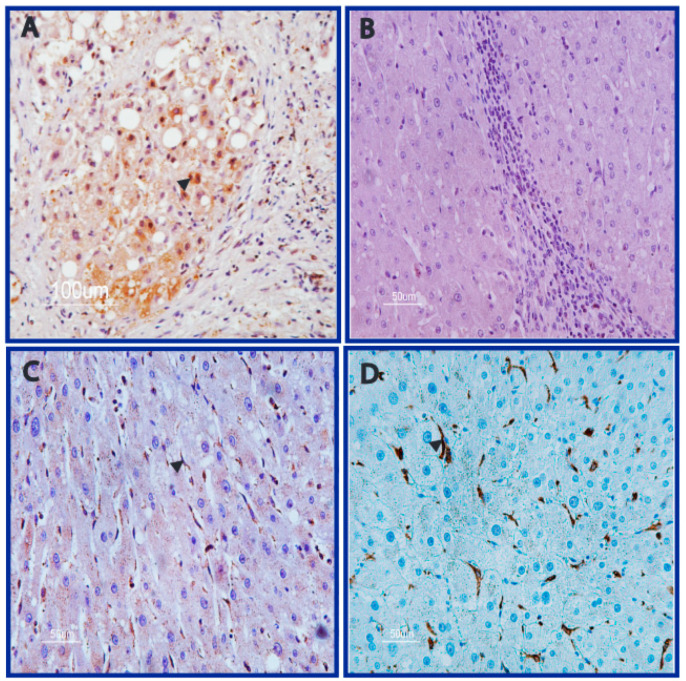
AFB1 staining in human cirrhotic tissue. Representative cases of AFB1adducts immunostaining in human cirrhotic nodules with AFB1 positive hepatocytes were surrounded by septa with positive cells (**A**). Conversely, septa surrounding negative nodules usually did not display AFB1 adducts (**B**). AFB1 adducts were observed also in infiltrating cells identified by CD68 immunostaining (**C**,**D**). Magnification: (**A**): 10×; (**B**–**D**): 20×.

Overall, 25 of 131 HCCs displayed AFB1-adducts in neoplastic tissue. AFB1-adducts were recognised both in neoplastic hepatocytes, and in cells infiltrating the tumor and non-tumor tissues, with variable degrees and extension. AFB1-adducts were more frequent at the cytoplasmic level, instead of at the nuclear level. Immunostaining was low (less than 10 cells) in 12 cases, intermediate (10–40 cells) in nine cases, and high (more than 40 cells) in the remaining four cases. Representative cases are shown in Figure 1A–D and Supplementary Figure S1A. Concerning cell types, six cases (four in the “high” group and two in the “intermediate” group) showed AFB1-adducts both in neoplastic hepatocytes and intratumoral infiltrating cells, while the remaining 19 cases showed AFB1-adducts mainly in intratumoral infiltrating cells (Figure 1E,F). These intratumoral cells were characterized by CD68 immunostaining, to confirm them as monocyte-derived macrophages which in the liver may be represented by Kupffer cells and tumor-infiltrating macrophages (Figure 1G,H). The matched cirrhotic tissue of HCC positive cases often displayed positive cells in the septa surrounding positive nodules. Conversely, septa surrounding negative areas did not display AFB1 adducts (Figure 2A,B). In line with HCC immunostaining pattern, AFB1 adducts in cirrhotic tissues were observed mainly in infiltrating cells identified by CD68 immunostaining, rather than in hepatocytes (Figure 2C,D). Among the 45 cases of CC, none displayed any immunostaining in tumor tissue. Similarly, among the 20 cases of end stage cirrhosis subjected to liver transplantation without HCC, only one showed a faint immunostaining in few infiltrating cells. Concerning HCC patients, no correlation was found between AFB1 adducts and etiology or clinical-pathological variables, except for a higher frequency in the female gender and a trend towards an association with mutated TP53 accumulation revealed by IHC assay. Remarkably, IHC detection of TP53 accumulation might underestimate the real extent of TP53 genetic lesions, since only accumulation of mutated TP53 can be detected, while TP53 deletion is not revealed by this assay. RFLP analysis of TP53 R249S transversion was performed in a subgroup of 77 patients (all 25 AFB1 positive cases and 52 AFB1 negative cases) on DNA extracted from HCC tissue. This genetic lesion was recognized in one case only (Supplementary Figure S1B), which was also characterized by a high AFB1 immunostaining in neoplastic hepatocytes and stromal cells and a high p53 accumulation in neoplastic hepatocytes. Exons 4-10 of the TP53 DNA sequencing was previously available for 27 matched HCC and cirrhotic tissues (7 in the AFB1 positive group and 20 in the AFB1 negative group) and included nine HCC mutated cases displaying IVS5-2A>C c.560-2A>C, IVS6+1G>A c.672+1G>A splicing mutations, p.R282W c.844C>T, p.R273L c.818G>T, p.R175L c.524G>T, p.S215R c.645T>G, p.A347V c.1040C>T missense mutations and p.W146X c.437G>A, p.R342X c.1024C>T nonsense mutations. Overall, four mutated cases belonged to the AFB1 positive group, while the remaining five belonged to the AFB1 negative group. Taken together, these results do not suggest any association between AFB1 adducts and TP53 mutation pattern, similarly to what previously described in low AFB1 exposed areas [15]. The smoking habit was known only in a subgroup of patients. Among the 25 patients with AFB1-adducts in HCC tissue, smoking history was known in 18 patients. Only one declared active smoking, while other three declared previous smoking (10–20 cigarette/day for two patients and 4–5 cigarette/week in one case) stopped more than 20 years before surgery. These four cases all displayed a faint AFB1-adduct immunostaining in noncancerous cells infiltrating HCC tissue. In the AFB1-adducts negative group, smoking history was available for 71 of 131 patients. Among these, 10 declared active smoking (10–20 cigarette/day in six cases and less than 10 cigarette/day in four cases) and two patients declared stop smoking respectively 15 and 20 years before surgery (about 20 cigarette/day in both cases). No significant association was found between smoking habit/history and the presence of AFB1-adducts. When combined, these findings confirm that AFB1-adducts may accumulate in the liver of patients with HCC–suggesting a possible contribution to HCC and not to CC–and are not related with actual or previous smoking habit.

### 3.2. DNA Adducts Are Present in AFB1-Exposed Rats HCC

**Figure 3:**
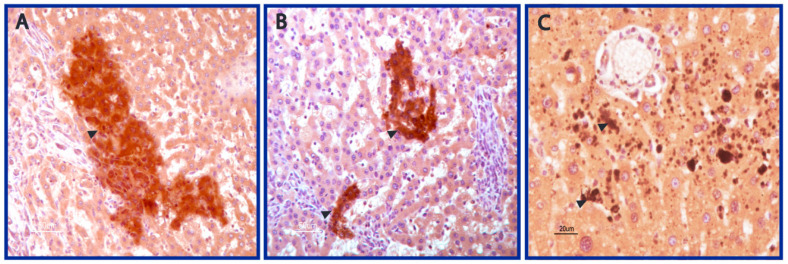
AFB1 staining in rat HCCs. (**A**–**C**). Strong AFB1 staining of neoplastic clusters and tumor-infiltrating cells in rat HCCs. Scale bars are reported. Magnification: (**A**,**B**): 20×; (**C**): 40×.

We next explored the six rats bearing both adenomas and HCCs, developed after AFB1 exposure to ascertain any morphological correlation with primary human HCC. As described for human HCC, a heterogeneity across rats’ adenomas and HCCs was observed in this setting too. Likewise in human HCC, both neoplastic hepatocytes and tumor-infiltrating cells displayed AFB1-adducts even if the inflammatory infiltrate is less represented in rat liver due to a fast carcinogenesis process and to the absence of hepatitis virus. Representative cases are shown in Figure 3A–C. As expected, due to acute exposition, the intensity of AFB1 staining was higher in neoplastic hepatocytes of rat HCCs compared to a human counterpart. Indeed, a similar staining in neoplastic hepatocytes was observed only in one case in the human setting, showed in Figure 1C. Remarkably, rat HCCs, adenomas, and cholangiocellular carcinomas developed spontaneously, or following benzene exposure did not display any immunostaining, confirming the specificity of the IHC reaction to AFB1-adducts. Taken together, these findings point to a similar mechanism of action of AFB1-adducts in AFB1-rat HCC and human HCC. A fainter IS was observed in most human cases, together with a prominence of positive cells in the non-hepatocytic compartment and a cytoplasmic localization in humans. 

## 4. Discussion

Food contamination by AFB1 is a worldwide concern, yet with heterogeneous geographical distribution. Climate changes and the globalization of alimentary habits may hamper a strict geographical definition of “high risk” areas. Indeed, the 2003 maize contamination revealed by rigorous food controls in Italy suggests also paying attention to AFB1 contamination in geographical areas considered at low risk. More recently, aflatoxins contamination was found in pistachio nuts, hazelnuts, almonds, and chili peppers marketed in southern Italy [16]. Due to this food contamination, we hypothesized that AFB1 adducts might be found in the liver of Italian patients. Thus, we investigated the presence of AFB1-adducts in the liver of an Italian cohort of patients with HCC, CC, or end-stage liver cirrhosis, from a low/chronic AFB1 exposure area. Overall, 25 of 131 HCC patients displayed variable levels of AFB1-adducts in their livers. Conversely, no CC and no cirrhotic liver without HCC displayed variable levels, except for one case of cirrhosis with a very faint immunostaining in few infiltrating cells. A rat model of AFB1-exposed HCC was also investigated to ascertain morphologic similarities with the human counterpart. Contrarily to previous reports [7,16], AFB1-adducts were recognized not only in hepatocytes, but also in tumor-infiltrating cells. This can be explained by the kind of AFB1 exposure in our cohort, which is expected to occur at low concentrations, maybe intermittently. Human HCC mainly displayed a cytoplasmic AFB1-adducts immunostaining in most cases. This pattern might be related with the localization of AFB1-adducts on mytochondrial DNA [17,18]. Remarkably, mytochondria may rely upon less efficient nucleic acids repair systems, and thus they are more likely to accumulate adducts on their own DNA and RNA. Beside this, it is also conceivable that low and intermittent exposure to AFB1 might lead to persistence of AFB1-adducts in phagosomes of hepatocytes, and even more relevantly, of tumor-infiltrating macrophages, because of debris and dead hepatocytes removal by phagocytosis. It is well known that macrophages and Kupffer cells–both recognized by CD68 immunostaining–phagocyte dead hepatocytes, and some residues may remain in their phagosomes for a long time. The presence of AFB1-adducts in hepatocytes and in tumor-infiltrating cells displayed the same pattern in human and rat HCCs, even though in the animal model the immunostaining was stronger and was more often both nuclear and cytoplasmic. As observed in human HCC, rat HCC displayed heterogeneous immunostaining patterns across different animals, emphasizing the lack of correlation between dose exposure and morphological changes, and suggesting that other factors, such as genetic polymorphisms of cytochrome p450 or detox efficiency, may contribute to AFB1-induced changes. As reported for human HCC, AFB1-adducts in rat HCC are present not only in DNA, but also, and at a higher level, in RNA [6] accounting for cytoplasmic immunostaining in this setting too. Even though our patient series is small, females still displayed a higher positivity for AFB1-adducts in their HCCs. This might be related to different alimentary habits, regarding higher consumption of tea and herbal teas as well as herbal dietary supplements, or to hormonal influences. However, these are pure speculations that need to be further investigated. No association was found between AFB1-adducts and cigarette smoking, even though these data were not available for all patients. We were surprised by the absence of AFB1 DNA-adducts in cholangiocarcinoma tissues. This might be related both to the absence of microsomal cytochrome P450 enzymes in biliocytes, making this cell lineage less exposed to AFB1 genotoxic hint, and to the very few infiltrating cells in our series of cholangiocarcinomas. Concerning the association between AFB1 adducts and TP53 immunostaining in HCC tissue (suggestive for TP53 mutation), we should remark that the IHC assay may underestimate the real extent of TP53 mutation, however, the numbers are too small to draw a definitive conclusion. The association between AFB1 exposure and TP53 mutation was previously ascertained by molecular studies addressing this specific issue [19]. In our series, TP53 R249S transversion was recognized in one case only, which also displayed a high AFB1 immunostaining in neoplastic hepatocytes and stromal cells and a high p53 accumulation in neoplastic hepatocytes. The association between TP53 R249S transversion and AFB1 adducts have been extensively assessed in focused studies, which confirmed its presence in 1% of HCCs from low exposure areas, compared to 7% in moderate exposure areas and 44% in high exposure areas, as reviewed by Smela et al. [20]. In addition, rats developing adenomas or HCC following AFB1 exposure do not display any mutation at the site corresponding to codon 249 of the human gene, underscoring the concept that the AFB1 and other risk factors such as HBV display a synergistic effect for this genetic lesion [21]. 

## 5. Conclusions

We report the presence of AFB1-adducts in tumor tissue of a non-negligible fraction of HCC patients from a low-exposure area such as Italy. Only few HCCs displayed a strong presence of AFB1 adducts, whereas most cases displayed less intense morphologic changes, suggestive of a possible intermittent and low-level exposure to aflatoxins. This does not prove a causal relationship with carcinogenesis; however, it is striking that no patient with end-stage liver disease without HCC displayed AFB1-adducts in liver tissue, except one with a faint positivity in few infiltrating/Kupffer cells. Remarkably, most patients with AFB1-adducts in HCC tissue displayed other risk factors for HCC, suggesting a contribution instead of a primary causative role of AFB1 to HCC development in our setting. Thus, at present, these data suggest to maintain a high attention on specific categories such as women, and to focus on detection and control measures of AFs to ensure food safety [3]; Ref. [11] also in geographical areas considered at low exposure. This preliminary series suggests to extend the investigation on larger populations of HCC patients from low AFB1 exposure areas, to exactly estimate the effects of a possible chronic and low-dose exposure in liver tissue, and to highlight the necessity of accurate food assessment on the presence of fungi and mycotoxin contamination. Future studies on larger series of AFB1-positive HCCs will investigate genetic lesions other than TP53 R249S transversion, trying to unravel the role of AFB1-DNA adducts in HCC not driven by HBV infection. Indeed, while the association between AFB1 and HBV infection has been deeply investigated at the molecular level, few data are available on HCCs associated with dysmetabolic diseases.

## Figures and Tables

**Table 1:** Demographic, risk factors and molecular associations.

	DNA-Adducts Positive	DNA-Adducts Negative	Significance
**Hepatocarcinoma (*N* = 131)**	**25/131 (19.1%)**	**106/131 (80.9%)**	**Chi-square, *p* = 0.048**
**male**	**16 (64%)**	**89 (84%)**	**Chi-square, *p* = 0.07**
**female**	**9 (36%)**	**17 (16%)**	**Chi-square, n.s.**
**TP53 accumulation**	**12 (48%)**	**29 (27.4%)**	
**Viral infection**	**21 (84%)**	**79 (74.5%)**	
**Non viral**	**4 (16%)**	**27 (25.5%)**	
HCV	14 (56%)	49 (46.2%)	
HBV	5 (20%)	17 (16%)	
HBV + HCV	1 (4%)	6 (5.7%)	
HBV + HDV	1 (4%)	0	
metabolic	3 (12%)	18 (17%)	
alcohol	1 (4%)	9 (8.5%)	
HCV + Previous HBV	0 (0%)	7 (6.6%)	
**Smoking history**	1/18 * (5.5%)	12/71 * (16.9%)	**Chi-square, n.s.**
**Cholangiocarcinoma (*N* = 45)**	**0**	**45**	
male		21	
female		24	
TP53 accumulation		14 (31%)	
Non viral		40 (88.9%)	
HCV		5 (11.1%)	
HBV		0	
**Smoking history**		8/29 *	
**Liver transplantation (*N* = 20)**	**1/20**	**19/20**	
male	1	13	
female	0	6	
TP53 accumulation	0	0	
HCV	1	11	
HBV	0	3	
HBV + HCV	0	1	
metabolic	0	3	
alcohol	0	1	
**Smoking history**	0/1	3/19	

* data available in a subgroup of patients.

## Supplementary Materials

The following supporting information can be downloaded at: https://www.mdpi.com/article/10.3390/nu14081652/s1, Figure S1: AFB1 immunostaining and TP53-RFLP.
