# Supplementary material for: Aflatoxin B1 DNA-Adducts in Hepatocellular Carcinoma from a Low Exposure Area
Source: Nutrients. 2022 Apr 15;14(8):1652. doi: 10.3390/nu14081652 (PMC9024438; doi:10.3390/nu14081652)

**Supplementary Figure S1. AFB1 immunostaining and TP53-RFLP.**

(A) Representative cases of AFB1-adducts immunostaining in neoplastic hepatocytes and tumor infiltrating cells displaying a variable degree and extension. Magnification: 40X. (B) Gel electrophoresis showing exon 7 mutation of p53 gene by PCR-RFLP using HaeIII. M, DNA ladder; 1,2,3,4 negative cases revealing 92 bp and 66 bp fragments denoting wild type p53. 5; positive HCC sample revealing uncleaved 158 bp, 92 bp and 66 bp fragments denoting heterozygous mutation.

Supplementary Figure S1

**A**

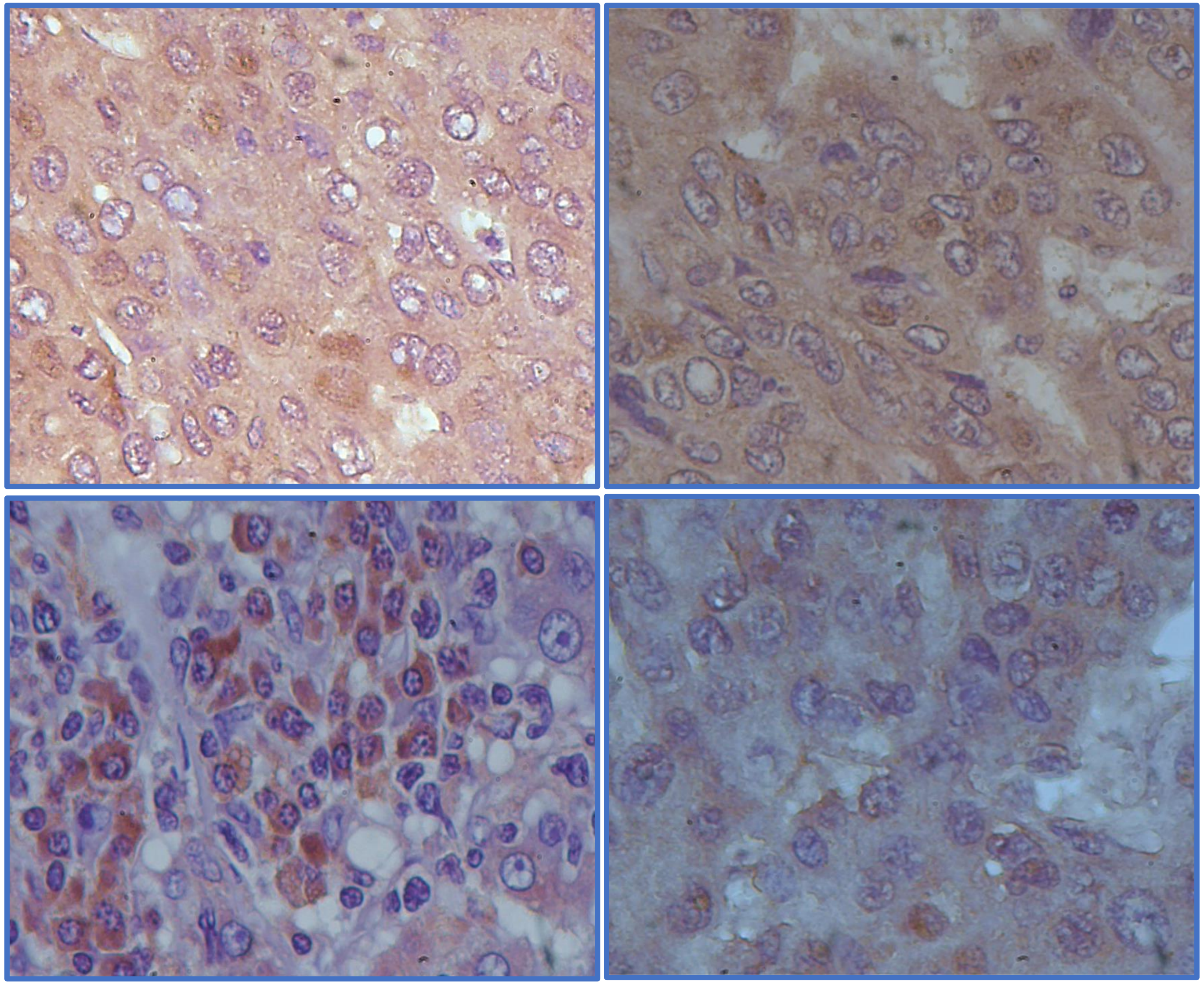

**B**

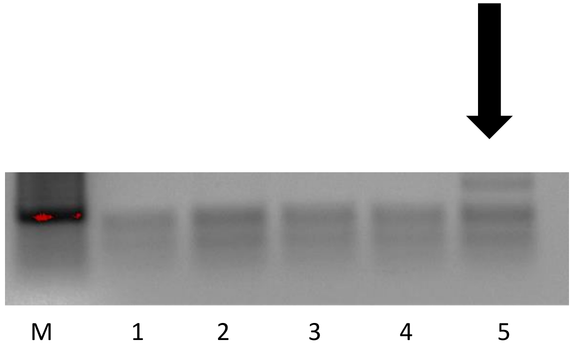

Supplement: Supplementary file 1 [file nutrients-14-01652-s001.zip › nutrients-1655328-supplementary.pdf]
